# Ivermectin compared with placebo in the clinical course in Mexican patients with asymptomatic and mild COVID-19: a randomized clinical trial

**DOI:** 10.1186/s12879-022-07890-6

**Published:** 2022-12-08

**Authors:** Carmen de la Rocha, Marco A. Cid-López, Blanca I. Venegas-López, Sandra C. Gómez-Méndez, Adriana Sánchez-Ortiz, Alma M. Pérez-Ríos, Ricardo A. Llamas-Velázquez, Aidé I. Meza-Acuña, Bárbara Vargas-Íñiguez, Daniela Rosales-Galván, Alejandra Tavares-Váldez, Nizdali Luna-Gudiño, Cinthia V. Hernández-Puente, Jovana Milenkovic, Cecilia Iglesias-Palomares, Miriam Méndez-del Villar, Gerardo A. Gutiérrez-Dieck, Carlos G. Valderrábano-Roldán, Jennefer Mercado-Cerda, Jocelyn G. Robles-Bojórquez, Arieh R. Mercado-Sesma

**Affiliations:** 1 Investigación Biomédica Para El Desarrollo de Fármacos S.A. de C.V. Zapopan, Tonalá, Jalisco México; 2 Hospital Regional de Zona 110, Instituto Mexicano del Seguro Social, Guadalajara, Jalisco Mexico; 3 Department Hospital Ángeles del Carmen, Hospitalization and Farmacovigilance Department, Guadalajara, Jalisco Mexico; 4 Centro de Investigación Multidisciplinaria en Salud, Centro Universitario de Tonalá, Universidad de Guadalajara, Av. Nuevo Periférico 555, Ejido San José Tateposco, CP45425 Tonalá, Jalisco México; 5 Departamento de Biología Molecular y Genómica, Centro Universitario de Ciencias de La Salud, Universidad de Guadalajara, Tonalá, Jalisco México

**Keywords:** COVID-19, Ivermectin, SARS-CoV-2, COVID-19 Treatment, COVID-19 clinical course

## Abstract

**Background:**

Despite the development and application of vaccines against Severe Acute Respiratory Syndrome Coronavirus 2 (SARS-CoV-2) around the world, the scientific community is still trying to find some therapies to avoid or ameliorate the fatal evolution of the Coronavirus disease 2019 (COVID-19). Since the publication of the potential use of ivermectin as a treatment against the disease, a pleiad of information about it has been published. However, the evidence is not strong or weak enough to conclude its usefulness in the clinical evolution of patients infected with SARS-CoV-2. We evaluate the efficacy and safety of ivermectin in the treatment of Mexican patients with asymptomatic and mild COVID-19 in a three-day administration in comparison to placebo.

**Methods:**

A randomized, double-blind, placebo-controlled trial was carried out in 66 adults with asymptomatic and mild COVID-19. Patients were randomly assigned 1:1 ratio to ivermectin plus acetaminophen or placebo plus acetaminophen. The primary endpoint was the proportion of subjects without a disease progression to severity according to COVID-19 guidelines by the National Institutes of Health (NIH) since randomization to 14 days.

**Results:**

None of the participants presented progression to a severe state in either group. Viral load was measured on Days 1, 5, and 14. No significant differences were observed in baseline or 14-day between groups (p = 0.720 and 0.362, respectively). However, on Day 5, a significant difference in viral load was observed between groups (p = 0.039). The frequency of symptoms was similar between groups, and no significant differences were observed. The most frequent symptom was cough. One severe adverse event associated with SARS-CoV-2 infection was observed in the ivermectin group.

**Conclusions:**

At standard doses, ivermectin is not effective to prevent progression to a severe state or reducing symptoms in adults with asymptomatic and mild COVID-19.

*Trial registration* The study was registered with ClinicalTrial.gov (NCT04407507) on May 29, 2020.

**Supplementary Information:**

The online version contains supplementary material available at 10.1186/s12879-022-07890-6.

## Background

Despite the development and application of vaccines against Severe Acute Respiratory Syndrome Coronavirus 2 (SARS-CoV-2) around the world, the scientific community is still trying to find therapies to avoid or ameliorate the fatal evolution of Coronavirus disease 2019 (COVID-19). Since the publication of the potential use of ivermectin as a treatment against the disease [1], much information about it has been published. However, the evidence is not strong or weak enough to conclude its usefulness in the clinical evolution of patients infected with SARS-CoV-2 [2]. Ivermectin has been described as a broad-spectrum antiviral, inhibiting nuclear import due to its ability to inactivate host nuclear transport proteins, such as integrase and NS5, limiting the ability of West Nile virus to infect at low concentrations [5]. It also inhibits the replication of yellow fever virus and other viruses, such as dengue, likely by attacking nonstructural helicase 3 activity [4].

The aim of the study was to evaluate the efficacy and safety of ivermectin in the treatment of patients with asymptomatic and mild COVID-19 in a three-day administration in comparison to placebo.

## Methods

### Study design and participants

The protocol was approved by the local ethics, biosafety, and investigation committees of the Investigación Biomédica para el Desarrollo de Fármacos S.A de C.V. and the Mexican health ministry Federal Commission for Protection against Sanitary Risks(COFEPRIS): 203301410A0055. The procedures were conducted in compliance with the Helsinki Declaration and Good Clinical Practice guidelines. Written informed consent was obtained from all patients. The study is registered on ClinicalTrials.gov: NCT04407507. The study adheres to Consolidated Standards of Reporting Trials (CONSORT) guidelines and includes a completed CONSORT checklist as an Additional file 1.

A randomized, double-blind, placebo-controlled trial was conducted to determine the efficacy and safety of ivermectin among subjects with asymptomatic and mild COVID-19. Subjects were included in two different sites in Guadalajara and Zapopan, Mexico: Hospital Hispano and Investigacion Biomedica para el Desarrollo de Farmacos (Ibiomed).

### Participants

Eligible participants were > 18-year-old men and women diagnosed with SARS-CoV-2 infection by real-time polymerase chain reaction (RT–PCR) testing of nasopharyngeal swab samples. We considered viral load undetectable when the threshold cycle (Ct) value of the nucleocapsid (N) gene from SARS-CoV-2 was ≥ 40. Patients with moderate or severe COVID-19 [9], diagnosis of other respiratory infections, impaired liver function tests (> 5 times above the normal level of alanine aminotransferase or aspartate aminotransferase), history of recurrent urinary tract infections, pregnancy or nursing women, active participation in other clinical trials, and use of antibiotics, verapamil, cyclosporine A, trifluoperazine or antiparasitic treatment for a concomitant disease were excluded. Moreover, subjects with a reported allergy or sensitivity to ivermectin, or acetaminophen, or its use during the protocol were also excluded.

### Randomization

Participants were randomly allocated 1:1 to receive oral ivermectin or placebo using a macro in Microsoft Excel (version 16.38; Microsoft Corporation, Redmond, WA, USA) with random numbers. The investigators or study coordinators enrolled and assigned participants to intervention. An unblinded pharmacist provided masked intervention according to permuted blocks of 2 in the randomization sequence. The rest of the clinical staff, investigators, and participants were blinded to the assignment.

### Interventions

Patients received 12 mg per day of ivermectin tablets or placebo for 3 days. Both groups received 500 mg acetaminophen tablets four times a day for 14 days to eliminate symptom bias. Ivermectin, Acetaminophen (Pharmacen, Laboratories Alpharma), and the placebo were provided by Ibiomed.

### Outcomes measures

The primary endpoint was the proportion of subjects without disease progression to severity according to COVID-19 guidelines by the NIH [9] from randomization to 14 days. Severity was considered if the participants had an oxygen saturation (SpO_2_) < 94% on room air at sea level, a ratio of arterial partial pressure of oxygen to fraction of inspired oxygen (PaO_2_/FiO_2_) < 300 mm Hg, a respiratory rate > 30 breaths/min, or lung infiltrates > 50%.

Secondary endpoints were the indirect analysis of the viral load using the threshold cycle (Ct) value of the nucleocapsid (N) gene from SARS-CoV-2 which is inversely related to the viral load, and the presence and frequency of COVID-19 symptoms were measured on Days 1, 5 and 14. Subjects were asked to complete a diary of symptoms and adverse events for 14 days. They recorded the presence of the following symptoms: fever, cough, muscular pain, fatigue, shortness of breath, headache, diarrhea, palpitations, expectoration, and “other”. In the “others” field question, several subjects answered hypogeusia/ageusia, hyposmia/anosmia, and back pain.

Vital signs (temperature, blood pressure, pulse rate, oxygen saturation, and respiratory rate) and RT–PCR were measured on Days 1, 5, and 14. Laboratory tests were performed at baseline and Day 14. A security follow-up phone call was performed on Day 21.

### Sample size and statistical analysis

The sample size was calculated according to the study by Wölfel et al. [10] We considered the viral RNA concentrations isolated in the throat and nasopharyngeal samples at the beginning of the infection and the difference of 10,000 copies at 10 days. A sample size of 54 patients provided 80% power to detect a 0.10 absolute difference in the proportions of the placebo group using a 2-sided test with a significance level of 0.05. The sample size was inflated to a total of 66 participants to allow for 20% dropouts.

An exploratory analysis was carried out to identify the nature of the variables and their distribution. All tests were 2-tailed. For the quantitative variables, Kolmogorov–Smirnov tests were used to identify whether they adjusted to the normality assumptions with a 95% confidence interval. The statistical analysis to compare the difference of means between groups was calculated with a t-test, and the ordinal variables were analyzed with the Chi^2^ test. Values of p ≤ 0.05 were considered significant. We used IBM SPSS software (version 26, IBM Corporation, Armonk, NY, USA) for all statistical analyses.

## Results

### Patients’ characteristics

**Fig. 1:**
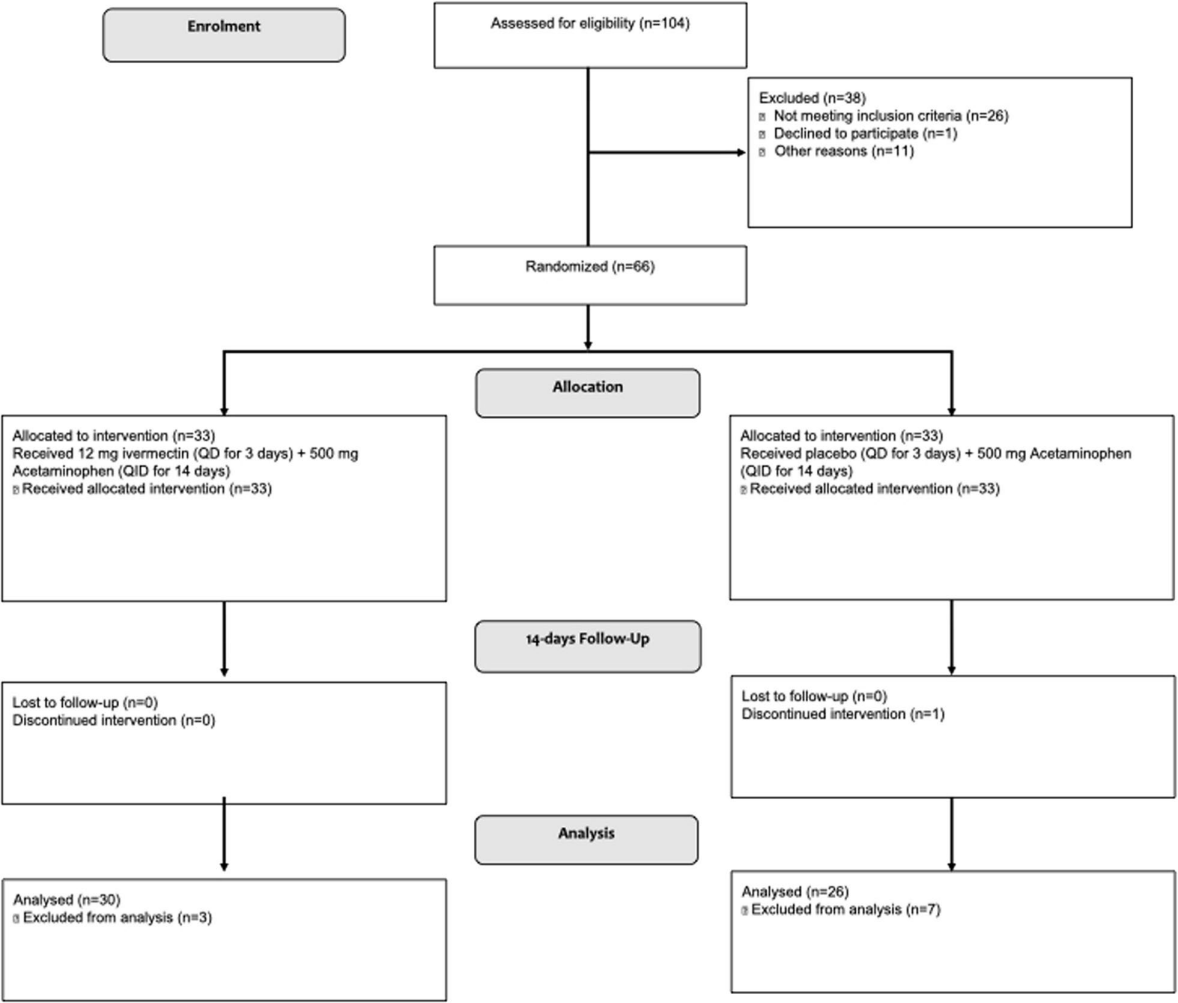
Enrolment, randomization, and follow-up of study participants

From 2020 July 21 to 2021 January 9, 104 subjects were screened, and 66 subjects were enrolled to receive either ivermectin (n = 33) or placebo (n = 33). Of these subjects, 10 were excluded from the efficacy analysis because nine of them were enrolled without a positive SARS-CoV-2 test, and one withdrew consent after the first visit (Fig. 1). However, they were included in the safety analysis.

The demographic and baseline characteristics of the group are listed in Table 1. The comparative analysis of the baseline measurements showed no differences between study groups.

**Table 1:** Demographics and baseline characteristics of the enrolled subjects (included in the efficacy analysis)

	Placebo		Ivermectin		P value
	(n = 26)		(n = 30)		
		95% CI		95% CI	
Sex, F/M (%)*	15/11 (58/42)		23/7 (77/23)		0.219
Age, years	36.4 (13)	31.3–5.1	40.4 (15.2)	35–5.4	0.229
BMI, kg/m^2^	26.5 (6.3)	24.1–2.4	27 (6.2)	24.8–2.2	0.494
Overweight, n (%)	9 (35)		10 (33.3)		0.920
Obesity, n (%)	4 (15)		7 (23.3)		0.455
Diabetes, n (%)	1 (3.9)		2 (6.7)		0.640
Hypertension, n (%)	1 (3.9)		4 (13.3)		0.214
Cardiovascular disease, n (%)	1 (3.9)		3 (10)		0.373
Hepatic disease, n (%)	2 (7.7)		0 (0)		0.122
Kidney disease, n (%)	1 (3.9)		0 (0)		0.278
BCG vaccination, yes (%)*	20 (77)		21 (70)		0.344
Smoker, n (%)	3 (12)		5 (16.7)		0.584
Vital signs					
Oxygen Saturation, %	95.7 (1.1)	95.3–0.4	95.9 (1.3)	95.4–0.5	0.323
Heart rate, bpm	73 (11)	68.8–4.2	82.2 (14.7)	76.9–5.3	0.154
Respiratory rate, bpm	18.5 (1.1)	18.1–0.4	18.4 (1.5)	17.9–0.5	0.384
Systolic pressure, mmHg	113 (15)	106.8–5.7	119.4 (21)	111.9–7.5	0.145
Diastolic pressure, mmHg	73.2 (10)	69.2–4	77.4 (11.8)	73.2–4.2	0.858
Body temperature, °C	36.4 (0.5)	36.2–0.2	36.4 (0.5)	36.2–0.2	0.349

Values in mean (SD)

F, female; M, male; BMI, body mass index; BCG, Bacille Calmette-Guerin

p < 0.05. * Pearson *χ*^2^ test

### Primary endpoint

None of the participants presented progression to a severe state in either group from baseline to Day 14.

### Viral load

**Fig. 2:**
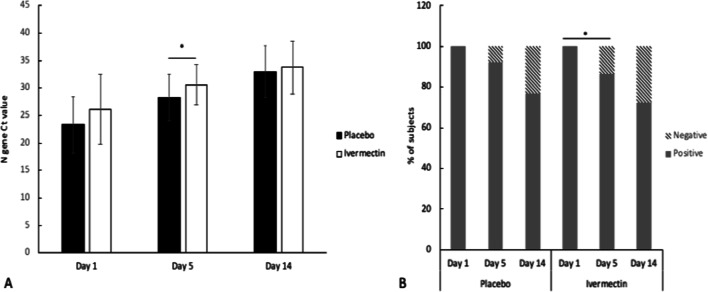
Analysis of the results of the SARS-CoV-2 diagnostic test by RT–PCR in subjects who received ivermectin and placebo on Days one, five and 14. **A** The Ct values are presented as the means (standard deviation) analyzed with Student’s t-test. **B** Diagnosis of the RT–PCR in percentage of subjects analyzed with * Pearson χ^2^ test. *, p < 0.05

The cycle threshold (Ct) value for gene N was measured on Days 1, 5, and 14, and no significant differences were observed between the placebo and ivermectin groups at baseline (23.3 ± 5.15 vs. 26.2 ± 6.36; p = 0.720) or 14 days (32.94 ± 7.74 vs. 33.74 ± 4.77; p = 0.362). However, on Day 5, a significant difference was observed between groups (28.25 ± 4.21 vs. 30.64 ± 3.74; p = 0.039) (Fig. 2A). On Day 5, viral load was undetectable in 13.3% of patients in the ivermectin arm and in 7.7% of patients in the placebo arm. On Day 14, the ivermectin-treated group reached a 28% negative rate, while 23% of the subjects treated with placebo presented negative results; no significant differences were observed between the two arms (p = 0.560). Both the ivermectin and placebo groups exhibited significant differences in the proportion of negative subjects between Days 1 and 14 (23% in placebo and 28% in ivermectin) (Fig. 2B).

### Symptoms

Compliance to the diary was 96% and 92.9% in the placebo and ivermectin groups, respectively (X^2^ = 2.23; p = 0.525). The data are presented as the percentage of days answered by each group.

**Fig. 3:**
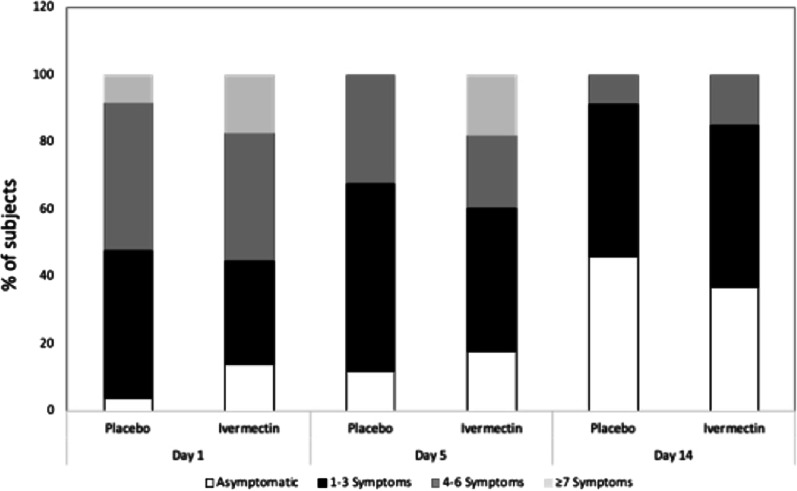
Symptom frequency in subjects who received ivermectin and placebo. Each bar corresponds to the percentage of subjects who reported the symptoms by day in placebo or ivermectin (white bars; asymptomatic; black bars 1–3 symptoms, grey bars 4–6 symptoms, and light grey ≥ 7 symptoms reported by day). From left to right are the values of days one, five and 14, respectively. X axis; percentage of subjects, Y axis; days and treatment. Pearson χ^2^ test

Cough, fatigue, myalgia, and headache were the most frequent symptoms. Regarding asymptomatic and symptomatic subjects (Fig. 3), on day one, 13.8% of the subjects who received ivermectin were asymptomatic, and 4% received placebo. At day five, asymptomatic patients accounted for 12% and 18% on placebo and ivermectin, respectively. On Day 14, these values reached 45.8% in those who received placebo and 37% in those who received ivermectin, and no significant difference was observed between groups regarding this distribution.

We also classified symptoms according to the number of symptoms per subject (1 to 3, 4 to 5, and ≥ 7 symptoms reported per day), showing the expected progressive decrease in the number of symptoms reported per day over time for both arms.

Regarding vital signs, the participants in the ivermectin group presented a higher heart rate on day 5 (72.78 ± 15.7 vs 83.46 ± 10.5 bpm; p = 0.007), but without a significant difference in comparison to baseline (p = 0.873) (Additional file 2: Table S1).

### Safety

Overall, 30 adverse events were reported, 17 in the placebo group and 13 in those treated with ivermectin. The majority of events were associated with COVID-19, but no differences were observed in the proportion of events that occurred between groups (Table 2). In the ivermectin group, a serious adverse event occurred; encephalitis secondary to SARS-CoV-2 occurred on study Day 10, and the subject made a full recovery. Regarding clinical laboratory tests, some differences were observed between baseline and Day 14 posttreatment: cholesterol and platelets increased in both the placebo and ivermectin groups. An increment in erythrocytes was observed from 5.05 ± 0.52 M/µL to 6.84 ± 2.60 M/µL (p = 0.002) from baseline to Day 14 (Additional file 2: Table S2).

**Table 2:** Adverse events reported in the study

Event	Placebo	Ivermectin	p value
Heartburn	1	0	0.652
Anxiety	0	1	0.348
Cystitis	0	1	0.348
Nasal congestion	2	0	0.201
Back pain	1	0	0.652
Encephalitis	0	1	0.348
Peptic acid disease	1	0	0.652
Exanthema	0	1	0.348
Pharyngitis	1	0	0.652
Gastritis	1	1	0.844
Acute gastritis	1	0	0.652
Hyperglycaemia	1	0	0.652
Arterial hypertension	1	1	0.844
Urinary tract infection	0	2	0.094
Leucocytosis	1	0	0.652
Elevated lipase	1	0	0.652
Low back pain	1	0	0.652
Dizziness	1	0	0.652
Nausea	1	2	0.713
Pneumonia	1	0	0.652
Right ear otitis	1	0	0.652
Maculopapular pruritus	0	1	0.348
Skin rash	1	0	0.652
Gastroesophageal reflux	1	0	0.652
Heat rash	1	0	0.652
Tachycardia	1	0	0.348
Vertigo	0	1	0.348

Values in frequency. Pearson χ^2^ test

## Discussion

In this clinical trial of patients with asymptomatic and mild COVID-19, those who were randomized to receive ivermectin for 3 days in the early stages of diagnosis at a dose of 12 mg/day presented a reduction on viral load within the five days after starting treatment in relation to the group that received placebo.

These results agree with those observed by Ahmed et al. [11], who evaluated the effect of ivermectin alone and in combination with doxycycline compared to placebo in hospitalized COVID-19 patients. They observed that treatment with ivermectin for 5 days resulted in earlier viral clearance (9.7 days) than the group treated in combination (11.5 days) or with placebo (12.7 days). A significant difference was observed against placebo at 7 and 14 days. In concordance, Pott-Junior et al. [12] found shorter times for obtaining two consecutive negative SARS-CoV-2 RT–PCR tests in subjects treated with ivermectin compared to those who received standard of care at hospital admission.

Chaccour et al. [13] published a pilot study of 24 patients, where they observed no difference in viral clearance after treatment with ivermectin at a single dose of 400 mcg/day or placebo in patients with mild symptoms. They reported that 7 days after treatment, all subjects remained with a positive SARS-CoV-2 test in the N gene; however, in both the N and E genes, a decrease in viral load was observed in those treated with ivermectin on days 4 and 7 post-treatment compared to placebo. These observations were accompanied by lower IgG antibody titters in the ivermectin group on Day 21 post-treatment. Similar results were reported in Lebanon by Samaha et al. [14] in a pilot clinical trial. They described a significant difference in Ct values between patients who received ivermectin compared to placebo from Day 0 to Day 3.

This effect could be associated in a directly proportional way to the dose, since a preliminary report indicated that providing ivermectin two times the dose that we administered (24 mg/day) for a single day translated into a higher proportion of subjects negative for COVID-19 at day five compared to 12 mg/day or placebo. In this trial, no statistical significance was observed; thus, it could be assumed that an indication of ivermectin for more days is required to reach such significance [15]. However, our study did not show a correlation between the calculated dose (12 mg between weight in kilograms) and the Ct value of the N gene (data not shown). According to Schmith et al. [16] report, single repeated doses of 200 mcg/kg, 120 mg weekly, or 60 mg every 72 h were not enough to reach the concentrations relative to the 50% inhibition (IC_50_) established by Caly et al. [3], but they did not intend consecutive daily doses as we did. The diverse results with similar or different posology could be explained considering the pharmacokinetics (PK) of ivermectin [17]. Absorption is intestinal, and diarrhea is a common clinical manifestation of COVID-19. This finding decreases the absorption rate and bioavailability and therefore the effect. Moreover, the elimination is principally in feces. However, there is no report of individual results of the effect or presence of diarrhea, and our data did not show any differences in Ct value between those who presented diarrhea and those who did not on any of the evaluated days (data not shown).

Similarly, a randomized study in hospitalized patients showed a benefit in the Ct value of patients treated with ivermectin for 7 days (100, 200, or 400 mcg/kg) in relation to those who did not receive ivermectin, with no-dose behavior.

Our findings and the others previously mentioned can potentially be translated into avoiding progression to severe disease. According to Liu's and Samaha's report, there is a relationship between the Ct value and the intensity of the disease [14]. Furthermore, other studies have shown that receiving ivermectin as part of COVID-19 treatment is associated with a lower mortality rate, accompanied by lower levels of inflammatory biomarkers, such as C-reactive protein, ferritin, and D-dimer [18].

In this study, an evaluation of the presence of symptoms associated with COVID-19 was carried out through the delivery of a diary; in general, the participants had an adherence to the completion of the symptom diary greater than 80%. The analysis revealed that the subjects who received ivermectin presented a higher frequency of symptoms from basal evaluation, of which fatigue and diarrhea were reported as adverse events expected from receiving ivermectin in the Food and Drug Administration (FDA) technical data sheet. In addition, in the same document, muscle pain and headache have also been observed in clinical trials [21]. Therefore, we could assume that these may be due to the drug and not the disease.

These findings do not correspond to the results by Chaccour et al. [13] in his pilot study, since they observed a lower frequency of the following symptoms in those treated with ivermectin: cough, anosmia, hyposmia, and shortness of breath. They did not observe any differences in the frequency of fever or an increase in the frequency of gastrointestinal symptoms by 3.5 times [11]. Recently, a randomized clinical trial by Shahbaznejad et al. [22] reported that patients who used ivermectin at a single dose, calculated according to weight, decreased the hospital stay and duration of symptoms compared to the control group.

On the other hand, a randomized trial with 400 patients, in which the time to resolution of symptoms was evaluated in subjects who received ivermectin (300 mcg/kg) for 5 days, did not observe a significant difference between groups, with a time to resolution slightly lower in the placebo arm (10 vs. 12 days) [23].

The evidence available to date seems to indicate that there is little or no benefit in the resolution of symptoms associated with the consumption of ivermectin at a standard dose [24].

Regarding safety issues, no relationship was found between the drug and any adverse event. The encephalitis event that occurred in the subject who received ivermectin was diagnosed as secondary to SARS-CoV-2 infection, a phenomenon that has been previously documented [25].

Taken together, these data do not show that ivermectin is effective in the treatment of COVID-19 by "accelerating" viral clearance in the first week, which may translate into a lower rate of complications. However, clinical trials with a larger number of participants are required at different doses and times of administration to elucidate the treatment with greater efficacy and a better safety profile. A limitation of the present study was the small sample size, especially for the interpretation of safety outcomes.

## Conclusions

At standard doses, ivermectin is not effective to prevent progression to a severe state or reducing symptoms in adults with mild COVID-19.

## Supplementary Information

**Additional file 1.** CONSORT checklist.

**Additional file 2.** Supplementary tables.

## Data Availability

The datasets used and/or analyzed during the current study are available from the corresponding author on reasonable request.

## Abbreviations

COVID-19: Coronavirus disease 19
SARS-CoV-2: Severe Acute Respiratory Syndrome Coronavirus-2
NIH: National Institutes of Health
COFEPRIS: Federal Commission for Protection against Sanitary Risks
CONSORT: Consolidated Standards of Reporting Trials
RT-PCR: Reverse Transcription-Polymerase Chain Reaction
SpO_2_: Peripheral oxygen saturation
PaO_2_/FiO_2_: Persistent arterial partial pressure of oxygen/fraction of inspired oxygen
Ct: Threshold cycle
RNA: Ribonucleic acid
bpm: Beats per minute
IC_50_: Inhibitory concentration
PK: Pharmacokinetics
FDA: Food and Drug Administration
