# Supplementary material for: Ivermectin compared with placebo in the clinical course in Mexican patients with asymptomatic and mild COVID-19: a randomized clinical trial
Source: BMC Infect Dis. 2022 Dec 8;22:917. doi: 10.1186/s12879-022-07890-6 (PMC9730611; doi:10.1186/s12879-022-07890-6)
Supplement: Supplementary file 2 — Additional file 2. Supplementary tables. [file 12879_2022_7890_MOESM2_ESM.docx]

**Additional file 2. Supplementary tables**

**Supplementary Table 1.** Vital signs in Days 1, 5 and 14.

| **Variable** | **Placebo** | **Ivermectin** | **p value** |
| --- | --- | --- | --- |
|  | **Day 1** | | |
| Oxygen saturation_,_ % | 95.8 (1.1) | 96.03 (1.5) | 0.339 |
| Heart rate, bpm | 74.38 (10.3) | 81.48 (14.65) | 0.151 |
| Respiratory rate, bpm | 18.71 (1.08) | 18.34 (1.44) | 0.314 |
| Systolic pressure, mmHg | 112.71 (15.09) | 118.32 (19.85) | 0.263 |
| Diastolic pressure, mmHg | 74.71 (11.37) | 76.64 (11.837) | 0.750 |
| Body temperature, ^o^C | 36.4 (0.51) | 36.45 (0.56) | 0.360 |
|  | **Day 5** | | |
| Oxygen saturation_,_ % | 96.2 (1.45) | 95.9 (1.4) | 0.943 |
| Heart rate, bpm | 72.33 (15.2) | 83.21 (9.5) | 0.283 |
| Respiratory rate, bpm | 21.05 (11.16) | 18.5 (1.7) | 0.081 |
| Systolic pressure, mmHg | 114.58 (18.6) | 112.8 (11.39) | **0.022*** |
| Diastolic pressure, mmHg | 73.29 (10.38) | 72.97 (8.72) | 0.501 |
| Body temperature, ^o^C | 36.34 (0.55) | 36.4 (0.30) | 0.769 |
|  | **Day 14** | | |
| Oxygen saturation_,_ % | 96.58 (1.6) | 96.07 (1.8) | 0.207 |
| Heart rate, bpm | 77.09 (9.3) | 81.4 (9.5) | 0.990 |
| Respiratory rate, bpm | 19.21 (1.6) | 17.7 (3.9) | 0.371 |
| Systolic pressure, mmHg | 112.5 (13.0) | 114.8 (12.1) | 0.559 |
| Diastolic pressure, mmHg | 74.4 (13.1) | 75.3 (9.54) | 0.297 |
| Body temperature, ^o^C | 36.16 (0.4) | 36.07 (0.5) | 0.444 |
| *Presented in mean (standard deviation) t-student test: *, P<0.05* | | | |

**Supplementary Table 2.** Laboratory findings on Days 1 and 14.

| **Variable** | **Placebo** | |  | **Ivermectin** | |  |
| --- | --- | --- | --- | --- | --- | --- |
|  | **Day 1  (n=26)** | **Day 14 (n=26)** | **p value** | **Day 1 (n=30)** | **Day 14 (n=29)** | **p value** |
|  | **Metabolic profile** | | | | | |
| Glucose, mg/dL | 100 (40.9) | 94.3 (23.2) | 0.379 | 93.4 (16.1) | 89.4 (13.8) | 0.315 |
| Ureic nitrogen, mg/dL | 12.4 (4.49) | 12.0 (3.73) | 0.960 | 11.9 (3.89) | 14.5 (10.9) | 0.225 |
| Creatinine, mg/dL | 0.72 (0.16) | 0.69 (0.13) | 0.456 | 0.81 (0.70) | 0.90 (1.23) | 0.732 |
| Uric acid, mg/dL | 4.66 (1.23) | 5.21 (1.68) | 0.318 | 4.11 (1.15) | 4.58 (1.18) | 0.133 |
| Total cholesterol, mg/dL | 163 (34.5) | 188 (55.2) | **0.048*** | 158 (35.2) | 186 (40.1) | **0.005**** |
| Triglycerides, mg/dL | 125 (57.7) | 147 (71.6) | 0.230 | 132 (48.9) | 154 (103) | 0.301 |
| HDL, mg/dL | 47.2 (12.2) | 51.2 (13.8) | 0.275 | 45.4 (13.0) | 49.8 (15.7) | 0.252 |
| LDL, mg/dL | 94.3 (32.3) | 113 (43.9) | 0.090 | 97.8 (33.4) | 114 (36.0) | 0.078 |
| VLDL, mg/dL | 22.8 (12.6) | 27.0 (15.7) | 0.300 | 22.3 (10.1) | 30.8 (28.0) | 0.121 |
|  | **Liver profile and electrolytes** | | | | | |
| Total bilirubin, mg/dL | 0.55 (0.19) | 0.633 (0.27) | 0.216 | 0.58 (0.29) | 0.65 (0.29) | 0.413 |
| Total proteins, g/dL | 7.29 (1.11) | 7.11 (0.49) | 0.460 | 6.70 (2.23) | 7.12 (0.38) | 0.318 |
| ALT, mg/dL | 40.0 (40.9) | 31.6 (24.3) | 0.076 | 30.4 (21.2) | 23.7 (11.1) | 0.134 |
| AST, mg/dL | 32.3 (23.9) | 44.1 (51.0) | 0.045* | 29.5 (24.2) | 20.8 (8.49) | 0.073 |
| GGT, mg/dL | 55.4 (72.6) | 54.2 (62.6) | 0.506 | 23.4 (14.3) | 24.3 (13.4) | 0.791 |
| Alkaline phosphatase, mg/dL | 88.0 (31.9) | 81.8 (25.0) | 0.077 | 73.3 (26.1) | 74.9 (23.5) | 0.810 |
| Sodium, mEq/L | 139 (2.48) | 139 (3.45) | **0.321** | 139 (3.31) | 139 (3.34) | 0.422 |
| Potassium, mEq/L | 4.04 (0.37) | 3.82 (0.86) | 0.205 | 4.05 (0.45) | 4.03 (0.44) | 0.854 |
| Chlorine, mEq/L | 104 (2.96) | 101 (20.8) | 0.136 | 103 (3.88) | 102 (18.3) | 0.727 |
| Magnesium, mEq/L | 2.11 (0.24) | 2.06 (0.33) | 0.530 | 1.99 (0.44) | 2.08 (0.39) | 0.386 |
| Calcium, mEq/L | 9.3 (0.48) | 9.46 (0.43) | 0.409 | 9.20 (0.51) | 9.51 (0.48) | 0.673 |
| Phosphorus, mEq/L | 3.73 (0.52) | 3.79 (0.49) | 0.664 | 3.57 (0.64) | 3.74 (0.83) | 0.381 |
|  | **Haematic biometry** | | | | | |
| Haemoglobin, mg/dL | 15.6 (1.57) | 14.1 (3.25) | 0.040 | 14.3 (2.22) | 14.0 (1.69) | 0.455 |
| Erythrocytes, M/uL | 5.05 (0.52) | 6.84 (2.60) | **0.002 **** | 4.71 (0.70) | 4.53 (0.51) | 0.291 |
| Haematocrit, % | 45.2 (4.46) | 41.06 (9.33) | 0.048 | 41.8 (6.21) | 40.8 (4.64) | 0.492 |
| Mean corpuscular volume, fL | 89.4 (4.46) | 83.75 (20.78) | 0.184 | 88.7 (5.46) | 89.4 (5.38) | 0.613 |
| Mean globular haemoglobin, pg | 33.4 (13.6) | 33.3 (20.08) | 0.354 | 30.8 (2.89) | 30.6 (2.44) | 0.772 |
| Mean globular corpuscular haemoglobin, g/dL | 34.5 (1.23) | 29.9 (12.0) | 0.065 | 34.2 (1.26) | 34.2 (1.10) | 0.861 |
| Platelets, k/uL | 247 (72.7) | 262 (86.60) | **0.001**** | 246 (85.3) | 294 (88.3) | **0.036*** |
| Leukocyte, k/uL | 6.72 (3.11) | 6.85 (3.25) | 0.138 | 5.82 (2.38) | 6.73 (2.42) | 0.152 |
| Lymphocytes, % | 30.8 (12.9) | 31.3 (10.9) | 0.332 | 35.0 (12.7) | 33.8 (11.1) | 0.700 |
| Presented in mean (standard deviation) t-student test: t-student; *, P<0.05; ** < 0.01. | | | | | | |
